# Virion encapsidated HIV-1 Vpr induces NFAT to prime non-activated T cells for productive infection

**DOI:** 10.1098/rsob.160046

**Published:** 2016-07-06

**Authors:** Kristin Höhne, Ramona Businger, Anouk van Nuffel, Sebastian Bolduan, Herwig Koppensteiner, Ann Baeyens, Jolien Vermeire, Eva Malatinkova, Bruno Verhasselt, Michael Schindler

**Affiliations:** 1 Institute of Virology, Helmholtz Zentrum München, German Research Center for Environmental Health, Neuherberg, Germany; 2 Heinrich Pette Institute, Leibniz Institute for Experimental Virology, Hamburg, Germany; 3 Institute of Medical Virology and Epidemiology of Viral Diseases, University Hospital Tübingen, Tübingen, Germany; 4 Department of Clinical Chemistry, Microbiology and Immunology, Ghent University, Ghent, Belgium; 5 HIV Translational Research Unit, Department of Internal Medicine, Ghent University, Ghent, Belgium

**Keywords:** HIV-1, Vpr, NFAT, productive T-cell infection

## Abstract

The majority of T cells encountered by HIV-1 are non-activated and do not readily allow productive infection. HIV-1 Vpr is highly abundant in progeny virions, and induces signalling and HIV-1 LTR transcription. We hence hypothesized that Vpr might be a determinant of non-activated T-cell infection. Virion-delivered Vpr activated nuclear factor of activated T cells (NFAT) through Ca^2+^ influx and interference with the NFAT export kinase GSK3β. This leads to NFAT translocation and accumulation within the nucleus and was required for productive infection of unstimulated primary CD4^+^ T cells. A mutagenesis approach revealed correlation of Vpr-mediated NFAT activation with its ability to enhance LTR transcription and mediate cell cycle arrest. Upon NFAT inhibition, Vpr did not augment resting T-cell infection, and showed reduced G2/M arrest and LTR transactivation. Altogether, Vpr renders unstimulated T cells more permissive for productive HIV-1 infection and stimulates activation of productively infected as well as virus-exposed T cells. Therefore, it could be involved in the establishment and reactivation of HIV-1 from viral reservoirs and might have an impact on the levels of immune activation, which are determinants of HIV-1 pathogenesis.

## 1. Introduction

The HIV-1 accessory proteins Vif, Vpu, Nef and Vpr are dispensable for HIV-1 replication in most immortalized cell lines but essential for viral replication *in vivo* [1]*.* They all mediate viral immune evasion and exert effects enhancing viral loads, but Vpr is still enigmatic. It is a 12.7 kDa small protein and consists of three amphipathic helices. It can form dimers and higher multimers, and is incorporated into progeny virions in high copy numbers [2]. Vpr has a modest positive effect on HIV-1 replication kinetics in some T-cell lines, activated primary CD4^+^ T cells and tonsil histocultures, as well as tissue macrophages [3–6]. Furthermore, enhancement of HIV-1 nuclear import and LTR transactivation, induction of G2/M-cell cycle arrest and apoptosis have been described in different cellular models [2]. However, until now, there is no link between the different Vpr effects and an essential *in vivo* function contributing to immune escape or high viral loads. Laguette *et al*. [7] suggested that Vpr promotes HIV-1 escape from immune sensing linked to G2/M arrest. However, work *in vivo* or evidence in primary cells for this hypothesis is not available. In humanized mice, Vpr mediated enhancement of CCR5 tropic HIV-1 replication in T_regs_ depleted this population, again associated with Vpr-induced G2/M arrest [8].

We initiated this study based on two hypotheses. First, because Vpr is the accessory protein with the highest abundance in the viral particle, we assumed that Vpr might exert its effects in the early phase of infection. Second, we aimed to investigate Vpr effects in host cells frequently encountered by HIV-1 *in vivo*: resting CD4^+^ T cells. Our data demonstrate the requirement of Vpr for efficient and productive infection of non-activated primary CD4^+^ T cells. Mechanistically, Vpr activates nuclear factor of activated T cells (NFAT) to achieve enhancement of non-activated T-cell infection by induction of Ca^2+^ influx and nuclear import of NFAT. Furthermore, we linked activation of NFAT by Vpr to induction of G2 arrest and LTR transactivation.

## 2. Results

### 2.1. Vpr mediates productive HIV-1 infection of non-activated primary CD4^+^ T cells

**Figure 1.**
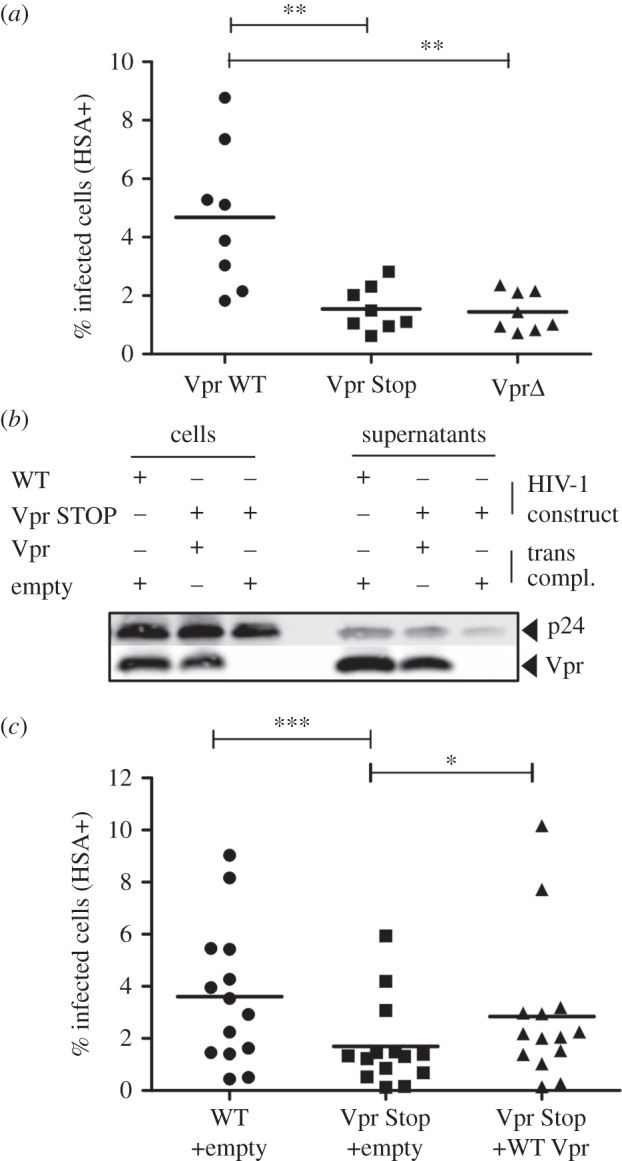
Vpr is required for the efficient infection of non-activated primary CD4^+^ T cells. (*a*) Freshly isolated CD4^+^ T cells were cultured in IL-2 to maintain viability and infected with HIV wild-type (Vpr WT), and point mutants mutated either at the start of Vpr (Vpr Stop) right after the Vpr start codon, also affecting the C-terminal Vif sequence, or late in Vpr (Vpr Δ) right after the Vif stop codon. Infection was done in the presence of Ritonavir for the detection of single round infection after 3 days by cell surface HSA staining (% infected cells). Results show the mean of eight independent experiments carried out on eight individual donors with independent viral stocks. (*b*) 293T producer cells were co-transfected with HIV WT or HIV Vpr Stop mutant vector and either an empty vector or one expressing Vpr. Cells and viral supernatants collected 2 days later were analysed by western blot for p24 and Vpr content. (*c*) Primary CD4^+^ T cells infected with virus as described in (*a*). Results show infectivity as a mean of 14 independent experiments carried out on 14 individual donors with independent viral stocks. Comparison to WT infection with Friedman test with Dunn's correction for multiple testing. *p < 0.05, **p < 0.01, ***p < 0.001.

To clarify if Vpr is capable of enhancing productive infection of non-activated primary CD4^+^ T cells, we used NL4-3-based infectious HIV-1 expressing non-signalling murine heat-stable antigen (HSA) at the surface as a marker of infected cells upon replication. CD4^+^ T cells were cultured in IL-2+ Ritonavir for single round infection, or additionally stimulated with PHA which resulted in a strong induction of activation measured by CD25 and CD69 (electronic supplementary material, figure S1). Then, we infected cells with HIV-1 variants expressing either intact Vpr or Vpr mutated with a stop codon right after the start codon (Vpr Stop). To exclude effects due to mutation of the overlapping Vif ORF, we constructed a HIV-1 HSA variant encoding stop codons after the Vif ORF, resulting in a virus that expresses Vif, but only a truncated Vpr protein (Vpr Δ, 23 N-terminal amino acids only). In unstimulated CD4^+^ T cells, WT Vpr HIV productively infected significantly more cells compared with the Vpr-defective variants Vpr Stop and Vpr Δ (figure 1*a*). By contrast, upon pretreatment of CD4^+^ T cells with phytohaemagglutinin (PHA) and therefore strong activation, the absence of Vpr did not result in a reduction of HSA-expressing cells (electronic supplementary material, figure S2). Thus, Vpr enhances the productive infection of non-activated CD4^+^ T cells.

### 2.2. Virion-delivered Vpr is sufficient to enhance productive HIV-1 infection of non-activated T cells

We next asked whether virus particle-associated Vpr can enhance productive infection rates of non-activated T cells or whether de novo synthesis of Vpr is necessary for this phenomenon. HIV-1 Vpr Stop was transcomplemented with Vpr and compared to uncomplemented virus. Importantly, Vpr content of transcomplemented HIV-1 Vpr Stop virions was comparable to parental WT HIV-1 (figure 1*b*). HIV-1 Vpr Stop transcomplemented with Vpr enhanced productive infection similar to WT HIV-1 (figure 1*c*). Of note, the effects observed did not stem from a Vpr-mediated effect on the initial HIV-1 entry efficiency in T cells, which we measured by determination of incoming p24 and the absence of de novo synthesized p55 Gag precursor (electronic supplementary material, figure S3). Furthermore, Vpr does not seem to influence the efficiency of nuclear import under these experimental conditions, because the amount of 2-LTR circles did not differ upon infection with WT or Vpr-defective HIV-1 variants (electronic supplementary material, figure S4). From these data, we conclude that virion packaged Vpr and its presence in the cell directly post-entry is sufficient to confer productive infection of non-activated CD4^+^ T cells.

### 2.3. Vpr stimulates NFAT activation in Jurkat T cells

Because Vpr is known to have a positive effect on transcriptional activation [2], we investigated the ability of Vpr to stimulate NFΚB [9,10] and NFAT activation [11,12]. Both are key regulators of transcriptional activation and also stimulate HIV transcription in the most important target cells for HIV-1 *in vivo*: CD4^+^ T cells and macrophages [13].

**Figure 2.**
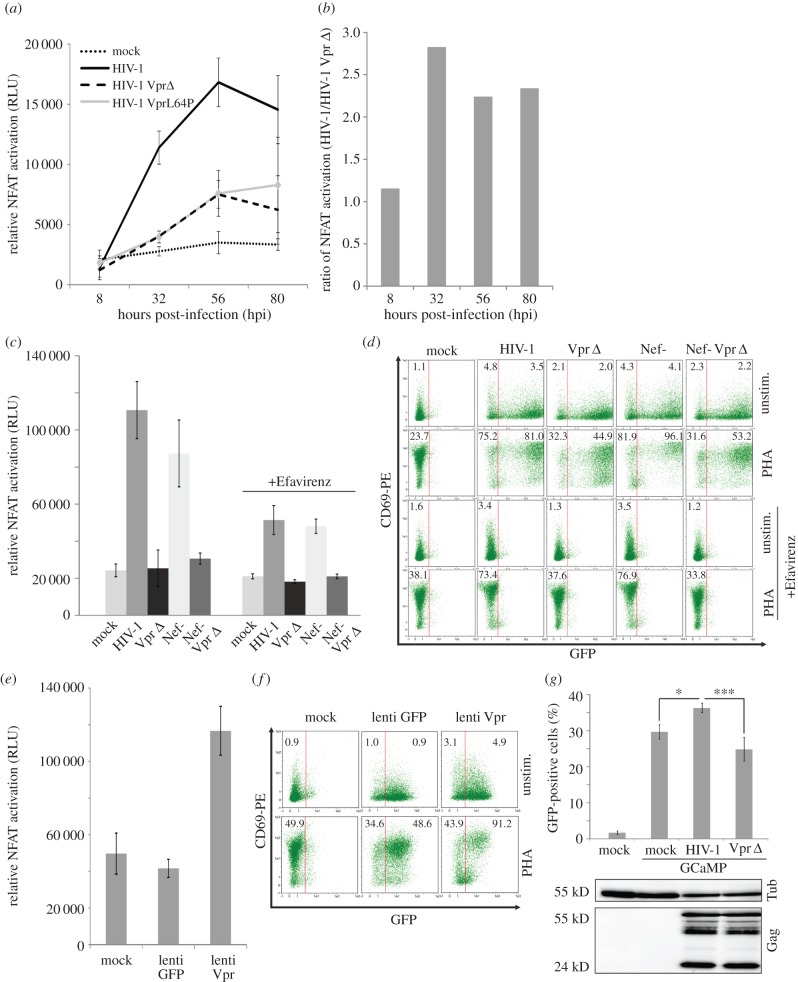
HIV-1 Vpr stimulates early NFAT activation. (*a*) 1 × 10^6^ Jurkat NFAT-luc cells were infected with 200 ng p24 of the indicated HIV-1 variants and PHA (1 µg ml^−1^) stimulated 8 h before each measurement to increase detection sensitivity of NFAT activity. Mean and standard deviation are of triplicates with two independent virus stocks from one representative out of three experiments. (*b*) Ratio of NFAT activation by WT HIV-1 versus Vpr-defective HIV-1. (*c*) Jurkat NFAT-luc cells were infected and measured for luciferase activity at 32 hpi as described in (*a*) with the indicated HIV-1 variants and treated with 100 nM Efavirenz to inhibit reverse transcription and viral gene expression. Mean and standard deviation are calculated from triplicate infections from one representative out of three independent experiments. (*d*) Aliquots from the Jurkat cells infected in (*c*) were stained for surface CD69 with a PE-labelled antibody. CD69-PE (early T-cell activation) and GFP expression as marker for productive HIV-1 infection were analysed by FACS. Indicated is the CD69-PE mean fluorescence intensity (MFI) of the GFP− or respectively GFP+ population of cells. We analysed two of the infection experiments described in (*c*) for CD69 and GFP expression with similar results. (*e*,*f*) Jurkat NFAT-luc cells were infected with Vpr-expressing lentivectors and subsequently luciferase activity (*e*) as well as CD69 surface and GFP expression (*f*) were assessed. Data in (*e,f*) are representative of three independent experiments. Numbers indicate CD69-PE MFI as explained in (*d*). (*g*) HeLa cells were transfected with the GCaMP5-GFP calcium sensor and infected 24 h later with 200 ng p24 of WT HIV-1 or the ΔVpr variant. Sixteen hours later, % of GFP+ cells was detected by FACS. Aliquots of the same cells were used to quantify p24 content by WB. Mean and standard deviation were calculated from three individual transfections and compared to WT infection with the one-way ANOVA with Bonferroni's multiple comparison post-test. *p < 0.05, ***p < 0.001.

In TNFα-treated and HIV-1-infected 293-reporter cells, we observed NFKB activation by Vpr only at the very late time points of infection (electronic supplementary material, figure S5). This suggests that Vpr-mediated NFKB activation is induced by *de novo* synthesized and not virion-delivered Vpr, at least in this experimental system. Contrarily, upon infection of Jurkat NFAT-luciferase reporter T cells with HIV-1 we observed time-dependent enhancement of NFAT activation (figure 2*a,b*). Thirty-two hours post-infection (hpi), NFAT activity in HIV-1-infected Jurkat cells was nearly threefold increased when compared with mock-infected cells. This phenotype was clearly attributable to functional Vpr expression, since Jurkat cells infected with a Vpr Δ HIV-1 or a variant expressing the Vpr L64P mutant, which is impaired for virion incorporation, showed reduced NFAT levels compared with WT HIV-1-infected cells (figure 2*a*). Of note, Vpr-dependent NFAT activation was not due to differences in infection levels between viruses (electronic supplementary material, figure S3; and data not shown). Nef and Tat also stimulate NFAT [14,15]. Hence, we expected the assimilation of NFAT activity between WT and Vpr Δ HIV-1 at later time points, when Nef and Tat are expressed (figure 2*a*,*b*).

To further assess whether Vpr is able to stimulate NFAT activation independent of Nef and Tat, we infected Jurkat NFAT reporter cells with HIV-1 variants devoid of functional Vpr and/or Nef expression and added the reverse transcriptase (RT) inhibitor Efavirenz to block reverse transcription and *de novo* production of viral proteins (figure 2*c*). At 32 hpi, Nef was not required to promote NFAT activation and Vpr clearly stimulated NFAT even when Jurkat cells were pretreated with Efavirenz. Additionally, as an independent marker for induction of T-cell activation, we measured cell surface expression of CD69 on productively infected (GFP+) as well as virus-exposed Jurkat (GFP-) T cells (figure 2*d*). Vpr enhanced CD69 expression in productively infected (GFP+) as well as the non-productively infected and virus-exposed population of cells (figure 2*d*), again suggesting that virion-delivered Vpr is sufficient to induce this phenomenon. Strikingly, Vpr also clearly enhanced CD69 expression in unstimulated Jurkat cells, suggesting that Vpr is able to directly induce T-cell activation.

Our data suggest that Vpr mediates early T-cell activation in HIV-1 infection. In order to show direct NFAT activation by Vpr, we performed independent experiments using a second generation lentiviral vector system allowing to transduce T cells without expression of HIV-1 proteins. Similar to HIV-1 infection, delivery and expression of Vpr by VLPs induced NFAT (figure 2*e*) and resulted in T-cell activation measured by expression of the early T-cell activation marker CD69 (figure 2*f*).

Previous reports show that administration of exogenous recombinant Vpr and Vpr peptides to purified mitochondria and cell lines increase calcium influx [16,17]. Since increased intracellular Ca^2+^ levels are part of the canonical pathway of NFAT activation [18], we hypothesized that Vpr might use this way to induce NFAT activation. To measure this, HeLa cells were transfected with the calcium sensor GCaMP, which activates GFP emission post calcium influx [19] and infected with WT or Vpr Δ HIV-1. Twenty-four hours later, flow cytometry demonstrated a higher percentage of GFP-positive cells and hence increased Ca^2+^ levels in the cells infected by WT HIV-1 versus the Vpr Δ variant (figure 2*g*). Importantly, equal Gag levels at that time point once again demonstrated independence of this phenotype from infection efficiency. Altogether, we conclude that virion-delivered Vpr is able to stimulate NFAT activation and induce T-cell activation early during HIV-1 infection possibly by raising the levels of intracellular calcium.

### 2.4. HIV-1 Vpr activates NFAT in unstimulated primary T cells and macrophages

Changes in intracellular Ca^2+^ levels should result in translocation of NFAT from the cytoplasm into the nucleus. Furthermore, to confirm experimental results from cell lines, we aimed to verify if Vpr is able to activate NFAT and enhance activation in primary target cells of HIV-1.

**Figure 3.**
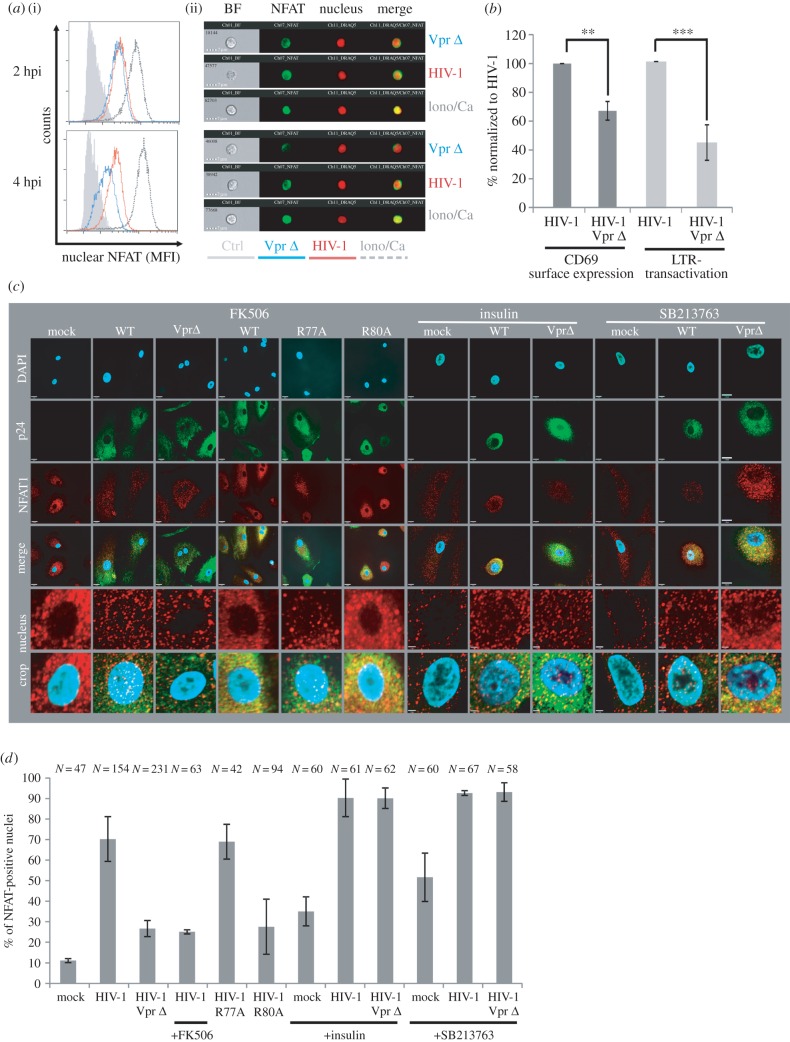
HIV-1 Vpr induces NFAT in primary T cells and macrophages. (*a*) ImageStream analysis of infected unstimulated CD4^+^ T cells, stained for NFAT and DNA, measured 2 and 4 h post-infection (hpi) with the viruses as indicated or stimulated with ionomycin. Histograms (i) show mean fluorescence intensity of nuclear NFAT staining, representative images are shown in (ii). (*b*) We used a previously established PBMC infection protocol mimicking effects on PBMC expressing low activation markers (see Material and methods) and measured CD69 surface expression (marker for early T-cell activation) in the GFP+, thus HIV-1-infected cell population and GFP mean fluorescence intensity (marker for LTR transactivation). Shown is the mean and standard deviation of three independent experiments with PBMCs from different donors, using two independent virus stocks. Unpaired Student's *t*-test (**p < 0.01, ***p < 0.001) and the Mann–Whitney test assuming non-parametric distribution (*p* < 0.05 for both parameters). (*c*) Macrophages were infected with 50 ng p24 of the indicated HIV-1 variants and treated with 10 ng ml^−1^ FK506 to inhibit NFAT activation as well as 100 nM insulin or 100 µM SB213763, both inhibitors of the NFAT export kinase GSK3β. Twenty-four hours later, macrophages were stained intracellularly for p24 (AlexaFluor 488), NFAT (AlexaFluor 555) and nuclei with DAPI for spinning disc confocal microscopy. (*d*) To measure NFAT activation, percentage of macrophages with NFAT-positive nucleus was manually counted in a blinded way (two to four different donors). *N* indicates the total number of analysed macrophages. Error bars show standard deviation.

We applied ImageStream analysis to visualize nuclear NFAT content. We infected freshly isolated unstimulated CD4^+^ T cells directly post-isolation from buffy coat with WT or Vpr Δ HIV-1 NL4-3. As shown in figure 3*a*, 2 h post-infection wild-type and Vpr Δ HIV-infected cells showed similar nuclear NFAT content. However, 4 h post-infection, nuclear NFAT content was lower in Vpr Δ HIV-infected cells compared with WT, illustrating Vpr is needed for sustained early T-cell activation. This was confirmed by phenotypic markers in T cells that are related to higher levels of NFAT: CD69 expression and HIV-1 LTR transcription [20]. We used an infection protocol of PHA prestimulated peripheral blood mononuclear cells (PBMC) previously established to achieve high productive infection rates in primary cells that phenotypically express low activation markers and hence allow to monitor effects of viral proteins on early T-cell activation [15]. Infections were done with the WT or Vpr Δ HIV-1 GFP reporter viruses, so that gating on GFP+ cells allowed to specifically measure CD69 expression in HIV-1 infected cells. Further, GFP is a readout for LTR activation, since it is expressed together with Nef from an LTR-driven bicistronic mRNA. In HIV-1 WT-infected PBMC, CD69 expression levels and LTR activation were 40–60% higher when compared with PBMC infected with HIV-1 Vpr Δ (figure 3*b*).

Although NFAT was described as transcription factor essential for T-cell activation [21], it is also expressed in macrophages in which the functional role is not entirely clear yet [22]. Primary monocyte-derived human macrophages (MDM) were infected with equal amounts of R5 tropic HIV-1 either with an intact Vpr ORF or Vpr Δ. We further infected MDM with HIV-1 containing a mutation at Vpr position R77A or R80A, known to have only a slight disruptive (R77A) or strong impairing (R80A) effect on HIV-1 replication in human lymphoid tissue and macrophages [5]. In non-infected MDM, NFAT localized mainly in the cytoplasm. By contrast, upon infection with HIV-1 (p24-positive cells), NFAT was predominantly present within the nucleus (figure 3*c*,*d*). This effect was Vpr-dependent, because after infection with Vpr Δ HIV-1 only a small proportion of infected macrophages showed NFAT translocation into the nucleus and we observed a similar phenotype when we used the calcineurin/NFAT inhibitor FK506 (Tacrolimus) to inhibit NFAT activation. In addition, NFAT translocation of the Vpr R77A variant was comparable to WT, whereas the R80A mutant showed a Vpr Δ phenotype (figure 3*c*,*d*).

GSK3β is an important nuclear export kinase for NFAT [18], and previously a mechanistic relationship between Vpr and the homologue of GSK3β in yeast Skp1 was suggested [23]. Therefore, we treated infected and non-infected macrophages with the GSK3β inhibitors insulin and SB213763 and monitored NFAT translocation (figure 3*c* right panels, *d*). Of note, inhibition of GSK3β upon HIV-1 infection resulted in nuclear NFAT localization in approximately 90% of cells, irrespective of functional Vpr expression. Hence, inhibition of GSK3β compensates for loss of Vpr. In conclusion, Vpr can induce NFAT activation in primary macrophages resulting in nuclear import of NFAT. A possible mechanism could be a Vpr-mediated interference with the activity of the NFAT export kinase GSK3β; however, we failed to show a direct interaction between Vpr and GSK3β (data not shown).

These data strongly suggest that Vpr is capable of inducing NFAT in primary unstimulated HIV-1 target cells (i.e. CD4^+^ T cells and macrophages).

### 2.5. Analyses of different Vpr mutants for their capability to stimulate NFAT activation, LTR transcription and G2 arrest

**Figure 4.**
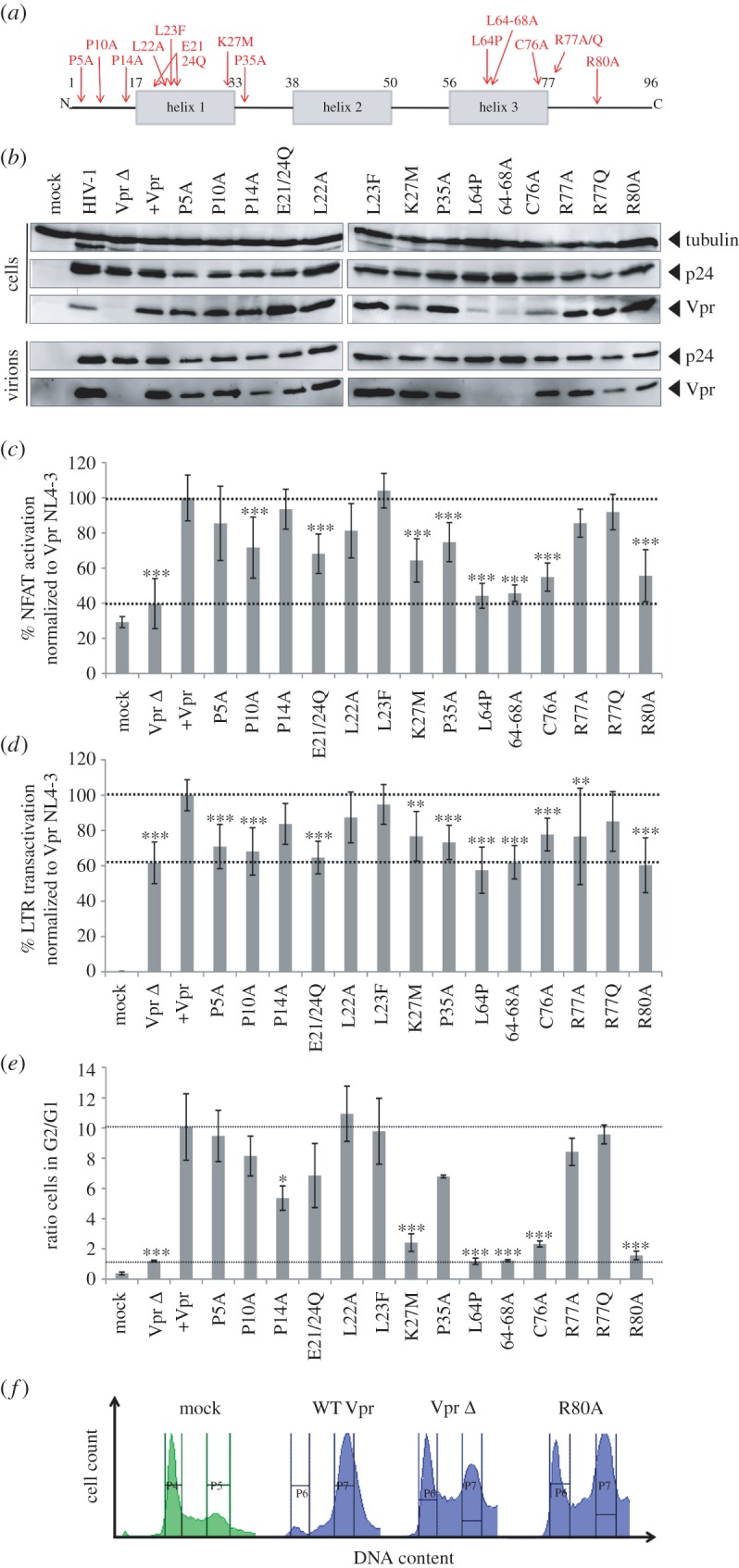
Induction of NFAT, LTR transactivation and G2 arrest by virion-delivered Vpr. (*a*) Schematic positions of the analysed Vpr mutants. Numbers give the respective amino acid position in NL4-3 Vpr. (*b*) 293T cells were transfected with HIV-1 NL4-3 Vpr Δ and cotransfected with plasmids expressing the indicated Vpr mutants. Lysates of the producer cells and the supernatants were harvested 36 h later and analysed by western blot. Shown is one representative out of two experiments. Ten nanograms p24 of HIV-1 Vpr Δ transcomplemented with Vpr and the indicated mutants (*b*) were used to (*c*) infect Jurkat NFAT-luc cells. Twenty-four hours post-infection, cells were stimulated with 1 µg ml^−1^ PHA and analysed for luciferase expression eight additional hours later. (*d*) CemM7 reporter cells expressing GFP under control of the LTR promoter were infected with transcomplemented HIV-1 Vpr Δ and GFP reporter activity was analysed by FACS 24 h later. Graphs in (*c,d*) depict mean values and standard deviation from four different experiments with triplicate infections. (*e*) Jurkat T cells infected with transcomplemented HIV-1 Vpr Δ coexpressing GFP were analysed for G2 arrest of GFP+ (i.e. HIV-1-infected cells 48 hpi). The graph depicts mean values and standard deviation of the ratio from the percentage of cells in G2 over G1 phase from three different experiments. (*f*) Representative DNA profile of GFP+ cells as in (*e*) either mock infected or infected with HIV-1 Vpr Δ transcomplemented with Vpr, the empty control plasmid or the R80A mutant. Gates indicate regions set to delineate cells in G1 and G2 phase of cell cycle. Statistical significance of the differences compared to WT infection in (*c*–*e*) was assessed with the one-way ANOVA with Bonferroni's multiple comparison post-test. *p < 0.05, **p < 0.01, ***p < 0.001.

To reveal putative functional correlations between different Vpr mutants and in order to get insights into the mechanism of Vpr-mediated NFAT activation, we initiated an in-depth analysis of a variety of established and previously described Vpr mutants (figure 4*a*) [5,24–26]. We transcomplemented Vpr (WT and mutants) into Vpr Δ HIV-1 during viral production allowing to monitor effects exclusively caused by viral particle-delivered Vpr. WB analyses revealed impaired expression and incorporation of Vpr L64P and 64-68A. By contrast, packaging of all other Vpr variants into HIV-1 particles was largely comparable (figure 4*b*).

Infection of Jurkat NFAT-luc cells showed a differential pattern of Vpr-dependent NFAT activation (figure 4*c*). The non-incorporated L64P and 64-68A were inactive in stimulating NFAT activity, underscoring that activity of virion encapsidated Vpr explains our observations. The proline mutations in the N-terminus of Vpr, as well as the changes introduced into the first half of the α-helix 1 had no or only slight effects on the levels of NFAT induced by Vpr. By contrast, the K27M mutant, although efficiently incorporated into viral particles (figure 4*b*), was attenuated. Similarly, and in accordance with the results obtained with HIV-1-infected macrophages [5], the well-incorporated R80A mutant could not stimulate NFAT activation, whereas the R77A/Q mutants showed a WT-like phenotype (figure 4*c*).

Vpr enhances HIV-1 LTR transactivation [27]. Because the LTR contains NFAT-responsive elements, we investigated this link using Vpr-transcomplemented HIV-1 viral stocks to infect CemM7 cells, expressing GFP under control of the LTR promoter [28]. Tat is the main transactivator of the LTR and therefore LTR activity was already high in the Vpr Δ infections (figure 4*d*). Nevertheless, 24 hpi Vpr-transcomplemented HIV-1 showed 40% increased LTR activity when compared with HIV-1 Vpr Δ. In general, and even upon exclusion of the two mutants which were not efficiently virion incorporated, mutants which were attenuated for NFAT activation were also impaired in their capability to enhance LTR transactivation. Hence, Vpr-mediated NFAT activation could be one determinant of Vpr's enhancing effects on the LTR.

Vpr-mediated induction of G2/M arrest is one of the best-investigated Vpr functions. Infection of Jurkat cells with Vpr-transcomplemented HIV-1 viral stocks also coexpressing GFP permitted quantification of cell cycle arrest specifically in the GFP+, hence infected cell population. Transcomplementation with WT Vpr resulted in a 10-fold induction of cells arrested in G2 in comparison to non-transcomplemented HIV-1 Vpr Δ (figure 4*e*). This is remarkable, since cells were analysed 48 hpi indicating that without de novo Vpr synthesis, virion-associated Vpr can have lasting effects in infected cells. In addition, most Vpr mutants defective for NFAT activation were also defective in induction of G2/M arrest (figure 4*e*; examples of primary FACS plots in figure 4*f*). Again, this phenotype is not explained by efficiency of Vpr virion incorporation, since Vpr C76A and R80A are efficiently incorporated, but lost their activity to promote G2 arrest as well as to induce NFAT activation, which is in contrast to the adjacent mutants R77A and R77Q.

### 2.6. NFAT activation by Vpr does not correlate with its subcellular localization, ability to induce PARP1 translocation, oligomerization or induction of apoptosis

**Figure 5.**
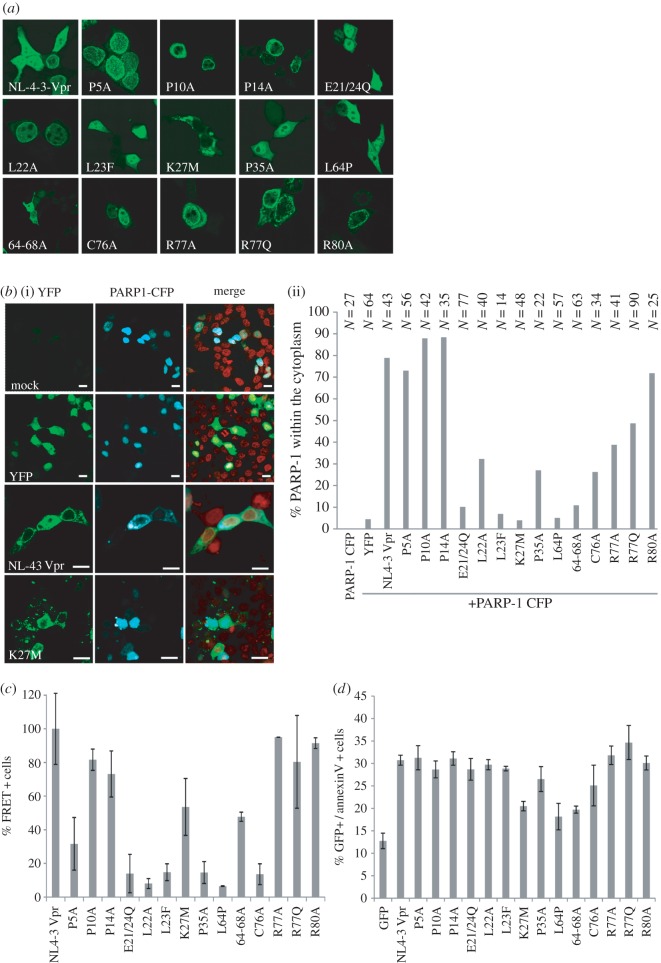
Localization of Vpr mutants and their capability to induce PARP1 translocation, homo-oligomerization and apoptosis. (*a*) Localization of the indicated Vpr-YFP mutants in transfected 293T cells. (*b*)(i) 293T cells were transfected to express the controls and the NL4-3 Vpr-YFP or the Vpr K27M-YFP mutant and cotransfected with PARP1-CFP. Twenty-four hours later, cells were stained with DRAQ5 and imaged by confocal microscopy. (ii) In 293T transfected to express PARP1-CFP and the Vpr-YFP mutants, PARP translocation was quantified by counting the percentage of cells with PARP1 in the cytoplasm. Samples were analysed in a blinded way. *N* is the number of cells analysed. (*c*) 293T transfected to express the CFP and YFP NL4-3 Vpr mutants were analysed by FACS-FRET to determine the extent of Vpr homo-oligomerization. Graph shows mean values and standard deviation from two independent experiments. (*d*) HeLa cells were transfected with vectors co-expressing Vpr or the respective Vpr mutants and GFP via an IRES, or GFP only as a control. Thirty-six hours later, cells were harvested and stained with AnnexinV-APC to measure apoptosis. The graph gives the absolute percentage of GFP+ AnnexinV+ double-positive cells. Mean values and standard deviation were calculated from three independent experiments.

Vpr has a variety of established *in vitro* functions including PARP1 translocation, oligomerization and induction of apoptosis [2,29], which might be linked to Vpr-mediated G2 arrest [30], virion incorporation [31] and/or NFAT activation [32]. We generated C-terminally YFP- and CFP-tagged fusion protein expression vectors of the different Vpr mutants allowing to investigate Vpr interaction with cellular factors and oligomerization by an FACS-based FRET assay [33]. As expected, NL4-3 Vpr-YFP localized to the nuclear rim, indicating that the YFP-tag does not interfere with intracellular sorting (figure 5*a*). Similarly, most other mutants showed this subcellular distribution. Exceptions were the E21/24Q, L23F and P35A mutants, with a more pronounced localization in the nucleus but also the cytoplasm; the K27M variant localized in large cytoplasmic accumulations and the L64P as well as the 64-68A mutants were diffusely spread throughout the cell (figure 5*a*). FACS-FRET of the different Vpr-YFP variants with a PARP1-CFP plasmid did not give a robust FRET signal and argued against a direct interaction of both proteins (data not shown). A larger complex involving also the glucocorticoid receptor could explain this [29]. Nevertheless, we confirmed translocation of PARP1-CFP from the nucleus into the cytoplasm upon Vpr-YFP cotransfection, indicating that there is indeed a functional interaction between both proteins (figure 5*b*(i)). Altogether, over the mutants tested, we observed no correlation of Vpr's activity on NFAT activation or G2/M arrest with PARP1 translocation (figure 5*b*(ii)), oligomerization of Vpr measured by FACS-FRET (figure 5*c*) and induction of apoptosis (figure 5*d*).

### 2.7. NFAT inhibition impedes Vpr-mediated LTR transcription, G2/M arrest, early T-cell activation and productive HIV-1 infection of resting T cells

**Figure 6.**
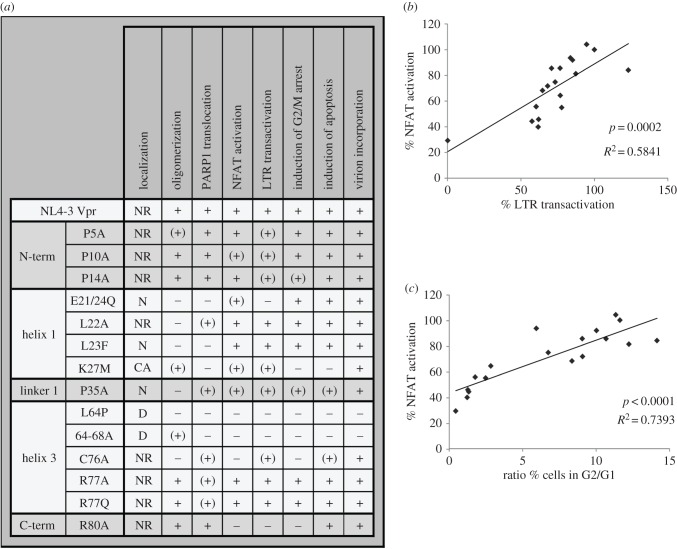
Overview and correlation of different Vpr functions. (*a*) Table showing the qualitative activity and functional features of the different Vpr mutants, ranked by structural region affected by the mutation (the data are directly derived from the experiments presented in figures 4 and 5). +, comparable to Vpr NL4-3; (+), attenuated; −, inactive; NR, nuclear rim; N, nuclear; D, diffuse; CA, cytoplasmic accumulations. (*b*,*c*) Correlation of Vpr-mediated NFAT activation with (*b*) LTR transactivation and (*c*) induction of G2 arrest. Every point represents the functional activity of one Vpr variant in the respective measurement (14 mutants, WT HIV-1, HIV-1 Vpr Δ, HIV-1 Vpr Δ transcomplemented with Vpr and mock, 18 data points in total).

The comprehensive analyses of different Vpr mutants suggested the presence of overlapping functional domains involved in the regulation of NFAT, LTR transactivation and G2/M arrest (figure 6*a*). Moreover, quantitative analyses of the mutant's activities revealed a significant and high correlation between NFAT activation and Vpr's ability to enhance LTR transcription and cause G2/M arrest (figure 6*b*,*c*). Thus, Vpr might activate NFAT to promote T-cell activation, arrest infected cells in the G2/M phase of the cell cycle, resulting in higher transcriptional activity, and therefore render resting T cells permissive for productive HIV-1 infection while enhancing LTR transcription.

**Figure 7.**
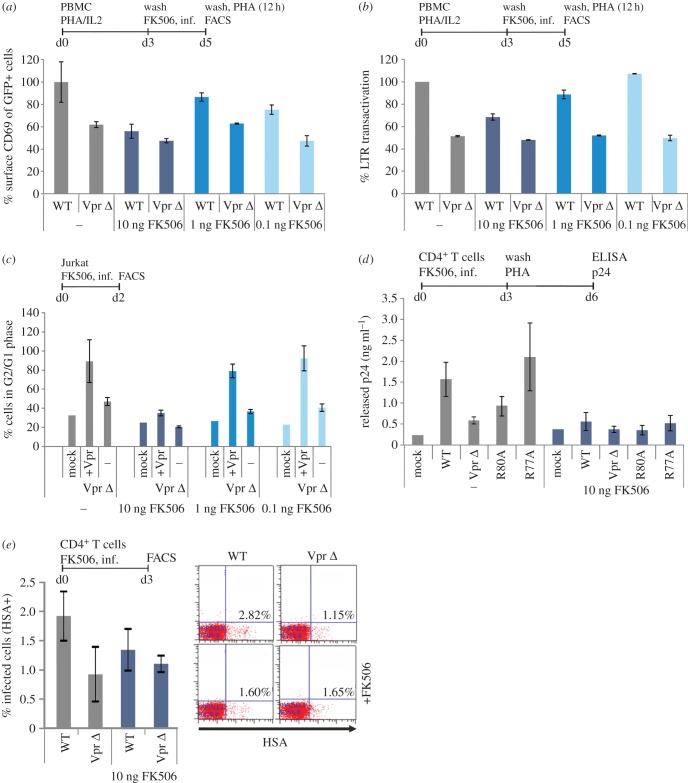
NFAT inhibition abrogates Vpr-mediated enhancement of T-cell activation, LTR transactivation, induction of G2 arrest and productive infection of resting T cells. (*a*) We used our previously established PBMC infection protocol mimicking effects on PBMC expressing low activation markers (see Material and methods) and added different concentrations of FK506 for 1.5 h prior to infection. Two days later, the PBMCs were washed and subjected to a PHA stimulus (1 µg ml^−1^). Twelve hours later, CD69 surface expression was quantified in the GFP+, thus HIV-1-infected cell population. (*b*) In PBMCs treated as described in (*a*), GFP mean fluorescence intensity was quantified as marker for LTR transactivation. In (*a,b*), mean values and standard deviation of two independent experiments with PBMCs from different donors that were infected in duplicates with independent virus stocks are shown. (*c*) Jurkat T cells were pretreated for 1.5 h with the indicated concentrations of FK506 and infected with the Vpr Δ HIV-1 IRES-GFP variant and the latter transcomplemented with Vpr. G2-arrest of the GFP+ HIV-1-infected cells was analysed 48 h post-infection. Graphs depict mean values and standard deviation from two different experiments with duplicate infections with independent virus stocks. (*d*) Unstimulated CD4^+^ T cells were preincubated for 1.5 h with 10 ng ml^−1^ FK506 and infected with 200 ng p24 of the indicated HIV-1 variants. Three days later, we washed the cells and added medium containing 1 µg ml^−1^ PHA to stimulate virus production from latently infected cells. Seventy-two hours later, supernatants were collected and virus production was measured by p24 ELISA. Mean values were calculated from infections of CD4^+^ T cells from one donor with two independent virus stocks. Shown is one representative out of five independent experiments. (*e*) Unstimulated CD4^+^ T cells cultured in the absence of exogenous IL-2 were treated for 1.5 h with 10 ng ml^−1^ FK506 or left untreated, washed and subsequently infected with HSA-expressing HIV-1 reporter viruses similar to the experiments presented in figure 1. Productive HIV-1 infection was measured by HSA staining 3 days later. The graph shows mean values and standard deviation of two infections. (*a*–*e*) The line bar above the diagrams concisely summarizes the experimental set-up indicating cells, stimuli, timing (d = day) and read out.

In order to explore this hypothesis, we performed infection experiments and incubated with different concentrations of FK506 during the infection period. We used this set-up because our data demonstrate that virion-delivered Vpr enhances NFAT activation early during the infection process. As hypothesized, when low levels or no FK506 was added prior to infection (figure 7*a*), the absence of Vpr resulted in reduced expression of the early activation marker CD69. Upon addition of FK506, CD69 stayed on a low level, regardless of functional Vpr expression. Similarly, FK506 treatment resulted in a dose-dependent reduction in the levels of Vpr-mediated enhancement of HIV-1 LTR transcription (figure 7*b*).

We then investigated the effect of FK506 treatment on Vpr-mediated G2/M arrest, similar to the experiment presented in figure 4*e*,*f*. Strikingly, with increasing concentrations of FK506, G2/M arrest induced by Vpr-transcomplemented HIV-1 Vpr Δ was reduced in a dose-dependent manner to levels similar to non-transcomplemented HIV-1 Vpr Δ (figure 7*c*).

Ultimately, we left freshly isolated unstimulated primary CD4^+^ T cells untreated or preincubated them with 10 ng FK506 (importantly, all in the absence of PHA and IL-2) and subsequently infected them with different NL4-3-based HIV-1 variants expressing Vpr mutants R80A, R77A or Vpr Δ. Three days later, T cells were extensively washed and stimulated with 1 µg ml^−1^ PHA to induce expression of latently integrated viral genomes and virus production (figure 7*d*). While we observed productive HIV-1 infection of resting T cells with WT HIV-1 or the Vpr R77A mutant, productive infection of resting T cells was lost upon infection with HIV-1 Vpr Δ or pretreatment with FK506 (figure 7*d*). Furthermore, when we infected unstimulated CD4^+^ T cells, cultured in the absence of exogenous IL-2, with the HIV-1 HSA reporter variants (figure 7*e*; compare with figure 1), we could demonstrate Vpr-dependent enhancement of productive HIV-1 infection and this Vpr effect was reduced by FK506 treatment. In sum, these experiments support a role for NFAT in Vpr-mediated infection of unstimulated CD4^+^ T cells.

## 3. Discussion

We show here that NFAT activation by virion-packaged Vpr is responsible for productive and enhanced HIV-1 infection of resting CD4^+^ T cells. Our data suggest that Vpr-activated NFAT is capable of inducing G2/M arrest, early T-cell activation and at least in part LTR transcription. The results further support a mechanistic model suggesting that Vpr increases intracellular Ca^2+^, which induces nuclear NFAT translocation. Consequently, Vpr leads to early T-cell activation and facilitates productive infection of resting T cells. Functional characterization of various Vpr mutants revealed a significant association between NFAT activation, the induction of G2/M arrest and enhanced LTR transactivation by Vpr. This correlation reflects a causal relation, because by inhibiting NFAT with FK506, Vpr-mediated T-cell activation, LTR transactivation and the G2/M-arrest in T cells was greatly reduced. Moreover, NFAT inhibition only during the early infection process abrogated Vpr-induced enhancement of resting T-cell activation.

Our results on Vpr-mediated NFAT activation were obtained with different cellular model systems and primary cells. We use HeLa cells, Jurkat T cells, differentiated monocyte-derived macrophages, unstimulated and prestimulated PBMCs, as well as non-activated and resting primary CD4^+^ T cells. Hence, the effects we describe are not restricted to certain cellular models or artificial immortalized cell lines but are relevant and confirmed in primary HIV-1 *in vivo* target cells*.* Furthermore, most experiments were done with fully complete infectious HIV-1 and with HIV-1 in which we transcomplemented Vpr into virions. Therefore, it is important to stress that virion-delivered Vpr is sufficient to induce all the phenotypes established. An early restriction to HIV-1 gene expression right after integration or in resting cells is the absence of the viral transactivator Tat. We hypothesize that Vpr has at least partly evolved to overcome this Tat deficiency in resting cells. An induction of even modest LTR transactivation will be sufficient to induce low levels of Tat, subsequently leading to efficient LTR transactivation and gene expression. Since the HIV-1 LTR contains different promoter elements, among others for NFAT and NFΚB, such a scenario is highly conceivable [20].

Our data revealed a correlation between Vpr-mediated NFAT activation and induction of G2/M arrest. Although our experiments are not yet sufficient to postulate a mechanistic relationship between these two functions, the data imply a connection between both Vpr activities. How could we explain such a relationship? Apart from regulation of various interleukins, NFAT modulates levels of cyclins and CDKs (cyclin-dependent kinases) [34,35]. A complex of cyclin B1 and p34Cdc2 controls the transition from G2 to M. NFAT might negatively regulate this complex during T-cell activation and this could suppress cell cycle progression [34]. Moreover, NFAT could promote transition into G2 by increasing the levels of cyclin A. This is part of a trimeric complex with CDK1 and CDK2 and regulates the changeover from S to G2 [36]. Furthermore, it was recently clearly demonstrated that virion-delivered Vpr is detected within the nucleus as soon as 45 min post-entry [37], explaining how Vpr could exert these effects in the nucleus early post-infection.

The aforementioned mechanisms together with the well-established role of NFAT in T-cell activation might promote a cellular environment facilitating the early steps of viral replication and enhancing productive HIV-1 infection. This phenotype is probably not observable in most immortalized T-cell line models of HIV-1 replication or even in *ex vivo* primary lymphocyte cultures, which need to be prestimulated to achieve HIV-1 replication. This would explain why previous studies failed to elucidate this Vpr phenotype. By contrast, one of the very few *ex vivo* systems permitting HIV-1 replication without exogenous stimuli is the human lymphoid tonsil tissue (HLT) culture system. In this experimental set-up, Vpr has a pronounced positive effect on viral replication and enhances IL-2 secretion [4,5], consistent with our data showing Vpr promotes early T-cell activation. Moreover, the mutation R80A disrupted Vprs' ability to augment viral replication in HLT, whereas the R77A/Q mutant replicated with WT-like kinetics [5], in line with our mutagenesis approach demonstrating loss of NFAT activation and NFAT-dependent enhancement of resting T-cell infection by the R80A change, but not by R77A/Q mutants. Of note, two independent studies also reported preserved interaction of Vpr R80A with SLX4 [7,38], a central component in a complex proposed to promote G2 arrest by Vpr. In addition, a recent study by the Planelles group found that interaction of Vpr with Mus81, another component of the SLX4 complex, is also dispensable for G2 arrest [39]. Hence, Vpr-mediated NFAT activation and the concomitant regulation of the cell cycle established by this study is most likely independent of Vpr's interaction with SLX4.

Vpr can moderately increase HIV-1 replication in another resting cell type: macrophages [5,40]. Although the role of NFAT is poorly understood in this cell type, NFAT can regulate the expression and secretion of various cytokines in macrophages including IL-6, IL-10, IL-12 and TNF-α [41]. IL-12 and TNF-α are proinflammatory cytokines potentially stimulating activation and hence HIV-1 replication in macrophages and T cells. IL-6 secretion promotes the recruitment of CD4^+^ T cells, which could contribute to HIV-1 transmission from macrophages to T cells. Of note, it was recently shown that Vpr increases TNF-α production from T cells [42], which independently supports our data and, more strikingly, the induction of NFAT by Vpr could be the mechanistic cause of increased TNF-α production.

Nuclear PARP1 represses NFAT activity [32]. We hence speculated Vpr-mediated PARP1 translocation from the nucleus into the cytoplasm as underlying mechanism of increased NFAT activation [29]. However, our mutagenesis approach argues against a functional relationship between both phenotypes. Our experiments revealed increased Ca^2+^ levels in cells infected with WT HIV-1 versus the ΔVpr variant. By contrast, previous results concerning Vpr and Ca^2+^ influx were obtained with exogenously administered recombinant Vpr and peptides thereof [16,17]. In conclusion, Vpr seems to activate NFAT via the canonical signalling pathway starting with increased calcium levels and activation of the NFAT dephosphorylating phosphatase calcineurin [18]. Vpr function could be substituted by inhibition of GSK3β mediated export of NFAT. However, extensive binding experiments including co-immunoprecipitation and FRET argue against a direct interaction of Vpr with NFAT or GSK3β (data not shown).

Comprehensive analysis of various Vpr mutants further revealed the independence of the NFAT phenotype from Vpr's subcellular localization, potency to form homo-oligomers and induction of apoptosis. In addition, oligomer formation was not required for efficient virion encapsidation and Vpr's capacity to induce apoptosis did not correlate with any of the other functions, except strong alterations in its subcellular localization.

Besides Vpr, HIV-1 Tat and Nef also increase NFAT activation [14,15], and it was previously suggested that Vpr potentiates Nef-induced NFAT activation [12]. While Vpr could indeed potentiate Nef-mediated NFAT activation, our data clearly demonstrate that Vpr alone is sufficient to induce NFAT in HIV-1-infected cells prior to viral gene expression. Furthermore, while previously Vpr expression plasmids were used and T-cell lines stimulated with PMA [12], our results are mainly based on HIV-1 infection experiments. Hence, we measure Vpr effects under physiological conditions and in primary HIV-1 target cells (i.e. macrophages and CD4^+^ T cells). Nevertheless, it is clear that Tat and Nef also positively influence late effects of NFAT activation (i.e. enhancement of T-cell activation and IL-2 secretion [43,44]).

What is the potential *in vivo* relevance of our work? Our data indicate that HIV-1 packages its own cellular activator into progeny virions. This finding is of importance to understand the initial steps of viral replication but also HIV-1 pathogenesis. For productive infection, proviral integration is not sufficient because the HIV-1 LTR promoter will be silenced in resting cells [45]. Low levels of Tat independent LTR promoter transactivation are essential to achieve first rounds of Tat expression and subsequent dramatic increase of Tat-mediated LTR transcription. *Ex vivo,* the initial Tat independent LTR transactivation is usually achieved by prestimulation of primary T cells with PHA or other mitogenic substances, leading to high levels of T-cell activation and cellular proliferation [15]. We demonstrate that Vpr sensitizes T cells for productive HIV-1 infection by activation of NFAT. In subsequent steps, Vpr in conjunction with Nef and Tat will contribute to cellular hyperactivation and therefore high levels of virus production. In this context, others have demonstrated that NFAT is an important factor of productive HIV-1 replication in primary T cells [20,46].

Intriguingly, Vpr is present in the plasma of HIV-1 infected patients and might penetrate non-infected cells by a protein transduction domain in its C-terminus [47]. Thus, Vpr might not only induce NFAT in infected cells but also lead to cellular activation in adjacent bystander T cells, priming them for productive infection. Considering the latter, Vpr could also be a viral factor contributing to chronic unspecific immune activation in HIV-1-infected subjects [48,49]. Because generalized immune hyperactivation is strongly associated with high viral loads, loss of CD4^+^ T cells and therefore AIDS progression [48], Vpr-mediated NFAT activation might be a determinant of HIV-1 pathogenesis. Accordingly, less pathogenic HIV-2 and most SIVs, which cause asymptomatic infections in their natural hosts, do not contain Vpr but express Vpx [50]. In addition, some long-term non-progressors were shown to carry Vpr-deleted virus [51]. Future studies investigating whether Vpx or related Vpr proteins from other lentiviruses are able to induce NFAT activation are highly relevant.

In summary, Vpr enhances productive infection of non-activated T cells through NFAT induction. By this mechanism, Vpr might also contribute to HIV-1 induced generalized hyperactivation of the immune system. This phenotype is associated with the emergence of high virus titres in the course of HIV infection. In this way, the data of this study add to the understanding of HIV-1-induced immune hyperactivation and the associated AIDS progression.

## 4. Material and methods

### 4.1. Plasmids and proviral constructs

R5 and X4 tropic pBR NL4-3-based constructs with deleted (pBR NL4-3 ΔVpr) or mutated Vpr genes [5] as well as the Nef-defective variants [52] were described previously. Splice overlap extension PCR was used to introduce the Vpr L64P mutation in the WT proviral backbone. Vpr pBR NL4-3 variants expressing an IRES-eGFP cassette to specifically identify the HIV-1-infected cell population were generated by subcloning of the Vpr ORF fragment via StuI and AgeI restriction sites into pBR NL4-3-IRES-eGFP [15]. Similarly, a Vpr- and Nef-defective IRES-eGFP variant was generated. HSA-expressing HIV Vpr Stop and Δ Vpr mutants were created by site directed mutagenesis on a part of the parental backbone, followed by substitution of the mutated fragment (with verified sequence) in the parental backbone NL4-3-IRES-HSA vector (kindly provided by Dr M. J. Tremblay, Faculté de Médecine, Université Laval, Québec, Canada), a vector expressing the truncated HSA marker from the Nef reading frame, together with Nef (HSA-IRES-NEF) [53]. To generate plasmids expressing Vpr and the various mutants as well as GFP via an internal ribosomal entry site (pCG vector) [54], splice overlap extension PCR was used to introduce the according changes into the Vpr ORF. Alternatively, Vpr was amplified from existing cDNAs. 5′ XbaI and 3′ MluI sites were introduced and used to ligate the fragments into the pCG-vector backbone. A similar strategy was used to generate CFP- and YFP-based Vpr fusion proteins in the pECFP and pEYFP vector backbone [33]. Here, 5′ NheI and 3′ AgeI restriction sites were used resulting in fusion proteins with the chromophore linked to the C-terminus of Vpr. Similarly, a PARP1-CFP fusion protein vector was constructed by amplification of PARP1 from a HeLa cell-derived cDNA. All PCR-derived inserts were sequenced to confirm nucleotide identity. Primer sequences used for PCR amplification and mutagenesis are available on request. Vpr expressing second generation lentiviral constructs pWPI-Vpr and controls [55] were kindly contributed by Eric A. Cohen (Montreal, Canada). Packaging plasmid psPAX2 and VSVG envelope pMD2G were received from Addgene.

### 4.2. Cell culture and virus stocks

293T and HeLa cells were maintained in DMEM or IMDM medium (Gibco BRL Life Technologies, Merelbeke, Belgium) and Jurkat E6.1, Jurkat NFAT-luc [14] and CemM7 [56] cells were cultured in RPMI (Life Technologies), all media containing the standard supplements. PBMC, CD4^+^ T cells and monocyte-derived macrophages (MDM) were generated from buffy coat (normal blood donors, Red Cross, Ghent, Belgium or Munich, Germany, blood donors gave written informed consent). CD4^+^ T cells were isolated by negative selection using paramagnetic beads (MACS; Miltenyi Biotec, Bergish Gladbach, Germany) or the Rossette Sep CD4^+^ T-cell isolation kit (StemCell Technologies, Grenoble, France). After isolation, the PBMC or CD4^+^ T cells were cultured in RPMI medium supplemented with 2 mM l-glutamin, 10% heat-inactivated fetal calf serum, 10–20 ng ml^−1^ IL-2 (Peprotech, Rocky Hill, USA), 100 U ml^−1^ penicillin, 100 g ml^−1^ streptomycin and depending on the experiment without or with phytohaemagglutinin (1 µg ml^−1^; Thermo Fisher Scientific, Waltham, USA), 3 days before infection with HIV-1. MDM were isolated and cultured as described [57].

HIV-1 viral stocks were produced by transfection of 293T cells with the proviral vectors. Transfection was performed with the calcium phosphate method or JetPei (Polyplus Illkirch, France) transfection kit according to manufacturer's instructions. To generate HIV-1 stocks with transcomplemented Vpr, the Vpr-defected NL4-3 backbones were cotransfected with the Vpr expression plasmid at a ratio of 4 : 1–20 : 1. To allow infection of CD4-negative 293T and HeLa cells a vesicular-stomatitis-virus G (VSV-G) protein expression plasmid was also cotransfected at a ratio of 20 : 1. We further pseudotyped HIV-1 with VSV-G to enhance infection efficiency of macrophages and PBMC and bypass possible effects of Vpr on viral entry. Viral supernatant was harvested 48 h after transfection and centrifugated at 900*g* for 10 min, to clarify the supernatant from remaining cells and debris. Vpr-expressing VLPs were generated as described before [55].

In all experiments, viral preps were rigorously normalized using reverse transcriptase activity or p24 content, and multiple batches of production were used. Concentration of the p24 antigen was measured in HIV-1 containing supernatant using the INNOTEST HIV antigen mAb ELISA kit (Innogenetics, Zwijnaarde, Belgium) or the p24 ELISA provided by the NCI (Frederick, USA) as described [58]. Alternatively, RT- activity values were determined using an in-house optimized and validated SG-PERT assay, and converted to p24 values [59].

### 4.3. Antibodies and other reagents

Antibodies used were against HSA (CD24): M1/69 fluorescein or phycoerythrin (BD Pharmingen, Erembodegem, Belgium) or allophycocyanin (Biolegend, San Diego, CA), CD69 (APC, Invitrogen Life Technologies, Darmstadt, Germany), HIV-1 Vpr (AIDS Research and Reference Reagent Program, Division of AIDS, NIAID, NIH, Germantown, MD, USA); HIV-1 Vpr (1–46) antiserum from Dr Jeffrey Kopp (catalogue #3951) or mouse anti-HIV-1 Vpr serum [25] (kindly provided by Ulrich Schubert, Erlangen), HIV-1 p24 (AIDS Research and Reference Reagent Program; Monoclonal Antibody to HIV-1 p24 (AG3.0) from Dr Jonathan Allan) [60] or HIV-1 p24 clone KC57-RD1 (PE or FITC conjugated, Beckman Coulter, Krefeld, Germany), mouse anti-tubulin (Sigma-Aldrich, Munich, Germany), rabbit anti-NFAT (CellSignaling Merck-Millipore, Schwalbach, Germany). Secondary antibodies were IRdye 800CW Goat anti-Rabbit IgG and IRdye 680LT Goat anti-mouse IgG (Li-Cor, Lincoln, USA), Alexa-555 donkey anti-rabbit and Alexa-488 goat anti-mouse (Invitrogen) and goat anti-mouse as well as goat anti-rabbit HRP (Dianova, Hamburg, Germany). Insulin and SB213763 were from Sigma-Aldrich (Munich, Germany) and FK506 from Invitrogen.

### 4.4. Western blot analysis

For western blot analysis, collected samples were stored in −20°C as dry pellet then lysed in 65% deionized water, 25% XT-sample buffer (Bio-Rad, Nazareth Eke, Belgium), 5% XT-reducing agent (Bio-Rad), 5% DTT. Lysed samples were spun through a Qiashredder column (Qiagen, Hilden, Germany) and boiled for 10 min at 95°C before loading on a Precarion pre-cast 12% Bis-Tris agarose gel (Bio-Rad). Gel was transferred to an Immobilion-FL membrane (Merck-Millipore) previously activated in methanol and blocked in Odyssey-blocking buffer (Li-Cor, Lincoln, USA) for 30 min. Primary antibody incubation (Vpr 1 : 2000, p24 1 : 2000, tubulin 1 : 2000) was done overnight at 4°C in blocking buffer +0.1% Tween. After three wash steps, secondary antibodies (1 : 5,000) were incubated for 60 min at RT in blocking buffer +0.1% Tween. Gel was visualized on the Odyssey infrared imaging system and band pixel intensity was quantified using the Odyssey software (Li-Cor, Lincoln, USA).

To assess expression and viral encapsidation of Vpr, 293T cells were transiently transfected with either pBR HIV-1 NL4-3 Vpr Δ complemented in *trans* with pCG-plasmids expressing either NL4-3 WT Vpr or mutants thereof, using CaCl_2_ transfection. Cells and virions were harvested 24 h post transfection, and cells were lysed in RIPA-buffer (1% NP-40, 0.5% Na-DOC, 0.1% SDS, 0.15 M NaCl; 50 mM Tris–HCl pH 7.4; 5 mM EDTA) for 5 min at 4°C. Subsequently, cell lysates were cleared by centrifugation at 12 000*g* and 4°C for 5 min. RIPA soluble proteins were separated in 12% SDS/PAA gels, according to Laemmli [61], transferred onto PVDF membranes (GE Healthcare, Munich, Germany) and probed with anti-tubulin, anti-Vpr or anti-p24 antibodies, followed by enhanced chemiluminescence detection. For internal controls, blots were stripped and re-incubated with the appropriate antibody. Additionally, cells and cell debris from virus containing supernatant were removed by centrifugation at 1000*g* for 5 min and 8000*g* for 10 min. Virions were in most experiments purified by centrifugation via 20% sucrose at 20 000*g* for 90 min, re-suspended in 1 ml of PBS, pelleted again for 20 000*g* for 90 min to remove serum albumins and finally analysed by Western blotting, using anti-Vpr (NIH) and anti-p24 (Abcam, Cambridge, UK) antibodies.

### 4.5. HIV-1 infection, virus collection and concentration

To obtain HIV-1-infected CD4^+^ T cells and CD4^+^ T-cell-produced HIV-1 supernatant, 40 ng of p24 antigen was added to 2.5 × 10^5^ cells and the culture was spinoculated at 500*g* for 90 min at 37 °C. After centrifugation, the supernatant was removed and the cells were further cultured in RPMI supplemented with 20 ng ml^−1^ IL-2. Three days after infection, phytohaemagglutinin, (1 µg ml^−1^; Thermo Fisher Scientific, Waltham, USA) was added for 3 days to the culture medium. Cells and supernatants were collected 7 days after infection, during peak of infection. The percentage of infected cells was determined by FACS analysis (MACSQuant Analyzer, Miltenyi Biotec, Bergish Gladbach, Germany) of HSA expression.

Collected viral supernatant was concentrated by ultracentrifugation. Briefly, viral supernatant was transferred to a polyallomer microcentrifuge tube and centrifugated in a Beckman L7-55 (rotors SW25.1 and SW50.1) at 70 000*g* for 30 min at 4°C to clarify the supernatant from remaining cells and other debris. Subsequently, supernatant was transferred to a new microcentrifuge tube and centrifugated at 210 000*g* for 90 min at 4°C. Supernatant was removed and concentrated virus was allowed to detach for 4–6 h.

Infection of unstimulated and stimulated CD4^+^ T cells for the measurement of WT and mutant HIV-1 virus infectivity was carried out 3 days after isolation by adding 60 ng p24 antigen to 2.5 × 10^5^ cells in the presence of the HIV protease inhibitor Ritonavir (AIDS Research Reference Reagent Program), throughout 1 µM in unstimulated and 10 µM in stimulated CD4^+^ T cell cultures to avoid multiple rounds of infection. The culture was spinoculated at 500*g* for 90 min at 32°C. After centrifugation, the supernatant was removed and CD4^+^ T cells were further cultured in RPMI supplemented with 20 ng ml^−1^ IL-2 and Ritonavir. Infectivity was measured by HSA reporter gene detection after 3 days of infection by FACS analysis (MACSQuant Analyzer). For infection of CD4^+^ T cells and PBMC with HIV-1 NL4-3-IRES-eGFP variants, 1 × 10^6^ cells were incubated for 6 h with 200 ng p24 of cleared virus stock in a total volume of 500 µl. Then cells were washed and 3 ml fresh media was added. Analysis of cell surface CD69 and GFP expression was done by standard FACS staining procedures in FACSCanto II (Becton Dickinson, Erembodegem, Belgium). Cells were fixed with 2% (m/v) PFA for 20 min prior to analysis. In all experiments, survival of infected cells was comparable as monitored by flow cytometry.

### 4.6. Analyses of intracellular Ca^2+^ levels using the Calcium sensor GCaMP

HeLa cells were transfected with the Calcium sensor GCaMP [19], and 24 h post transfection cells were infected either with VSV-G pseudotyped WT HIV or HIV-1 ΔVpr. Twenty-four hours post-infection, intracellular GFP expression was analysed by flow cytometry. In addition, cells were lysed in 0.5% Triton lysis buffer (150 mM NaCl, 50 mM Tris–HCl, 0.5% Triton X-100 and a protease inhibitor cocktail; Roche). Cell lysates were cleared by centrifugation at 10 000 r.p.m. and 4°C for 5 min. Soluble proteins were separated in 12% SDS/PAA gels and transferred onto PVDF membranes (GE Healthcare) and probed with specific antibodies, followed by enhanced chemiluminescence detection. For internal controls, blots were stripped and re-incubated with the appropriate antibody.

### 4.7. Measurement of 2-LTR circles, G2/M arrest, apoptosis, Vpr oligomerization and luciferase activity

2-LTR circles were measured according to a previously established protocol modified to a digital droplet PCR platform (Bio-Rad) [62]. To assess G2/M arrest, 48 h post-infection Jurkat E6.1 were pelleted at 150*g* and washed with PBS. Cells were fixed for 20 min with 2% (m/v) PFA, washed again and permeabilized by dropwise addition of ice cold 80% (v/v) ethanol and incubation for at least 1 h. Then cells were centrifuged at 150*g* for 5 min and resuspended in 200 µl of propidiumiodide (PI) solution (50 mg ml^−1^ PI and 0.33 mg ml^−1^ RNase A in PBS). After 30 min of incubation at 37°C, cells were analysed in a FACSCanto II (Becton Dickinson).

To assess Vpr-induced apoptosis, HeLa cells were transfected with the pCG-Vpr expression plasmids using Lipofectamine 2000 (Invitrogen) as recommended by the manufacturer. Thirty-six hours post transfection cells were trypsinated and stained with 5 µl AnnexinV-APC (Biolegend) in 100 µl staining buffer for 15 min at RT. Cells were then directly analysed by FACS with FACSCanto II instrument (Becton Dickinson).

Vpr oligomerization was investigated by FACS-based FRET analysis [33]. In brief, 150 000 293T cells were seeded in 12-well plate and transfected with 1.25 µg DNA of each the Vpr-eYFP and corresponding Vpr-eCFP fusion protein expression plasmid by the CaCl_2_ technique. Media was changed 6 h later and cells were harvested for FACS analysis (FACSCanto II, Becton Dickinson) 24 h later. The gating strategy to exclude false-positive FRET signals and assess FRET in the double-positive cell population is described elsewhere [33].

Luciferase activity of Jurkat NFAT-luc cells [14] and 293 NFKB cells was measured with the Promega luciferase reporter assay 8 h post-stimulation with 1 µg ml^−1^ PHA (NFAT) or 10 ng ml^−1^ TNFα (NFKB), respectively. For NFAT measurements, aliquots containing 50 000 Jurkat NFAT-luc cells were transferred in V-shaped 96-well plates and pelleted by centrifugation (5 min, 150*g*). Subsequently, cells were lysed with 25 µl lysis buffer for 20 min at 4°C. Then 20 µl cell lysate was transferred in white-walled plates and immediately measured after addition of 40 µl luciferase substrate in an Infinite M200 (Tecan, Männedorf, Switzerland) or Biotek Cytation 3 multiplate reader. Alternatively, an in-house luciferase assay was used with 100 000 cells, lysed in 60 µl. Forty microlitres of lysate were transferred to 40 µl assay buffer (100 mM KPO_4_ pH 7.8, 15 mM MgSO_4_, 4 mM ATP in H_2_O) and 40 µl substrate (15 mg Luciferin ml^−1^ assay buffer). Integration time was 100 ms per well. For assessment of NFKB activation, ten thousand 293 NFKB cells were seeded per 96-well plate, infected with equal nanogram p24 amounts and measured as described above.

### 4.8. Confocal microscopy and immunofluorescence

Translocation of PARP1 by Vpr was investigated by cotransfection of the Vpr-YFP fusion protein vectors and the PARP1-CFP construct in 293T cells seeded on coverslips. Twenty-four hours later, cells on slips were washed, fixed with 2% (m/v) PFA for 15 min and embedded with Mowiol 4-88 (Carl Roth) mounting solution on objective slides. DRAQ5 was added at a concentration of 1 : 1000 to the Mowiol to stain the nuclear DNA. Slides were allowed to dry overnight in the dark and then analysed on a CLSM Zeiss LSM510 with metadetector or a Nikon TiEclipse with the UltraViewVox Spinning Disc system (Yokogawa CSU-X1 and PerkinElmer, Waltham, MA, USA).

For analysis of NFAT translocation in human macrophages, 1 × 10^5^ cells were grown on coverslips and infected with 50 ng p24 of the HIV-1 viral stocks. Twenty-four hours post-infection, cells were washed with PBS and fixed with 2% (m/v) PFA for 20 min at 4°C. Macrophages were permeabilized with 1% Saponin for 15 min at RT and subsequently blocked for 20 min with 5% BSA (m/v) in PBS. Primary antibody staining was a 1 : 50 dilution of NFAT-specific antibody (CellSignalling) in 1% BSA/PBS and the anti-p24 antibody (also 1 : 50, clone KC57 Beckman Coulter) for 3 h at RT. Secondary antibody staining was done after PBS washing with anti-mouse Alexa-488 and anti-rabbit Alexa-555 (1 : 500 dilution in 1% BSA (m/v) in PBS) for 1 h at RT. Nuclear DNA was finally stained by 15 min RT incubation with DAPI solution (1 µg ml^−1^). Then cells were mounted with Mowiol on objective slides and imaged with a Nikon TiEclipse microscope as described above.

### 4.9. Imagestream analysis

CD4^+^ T cells were directly isolated from buffy coat by the Rossette Sep CD4^+^ T cell isolation kit (StemCell Technologies, Grenoble, France). Then, 1 × 10^6^ T cells were either stimulated with 1 µM Ionomycin/2 mM CaCl_2_ or spinoculated for 30 min at 4°C in 48-well plates with 500 ng p24 of HIV-1 WT or Δ Vpr pseudotyped with VSV-G to obtain high infection rates. Then cells were shifted to 37°C and incubated for 2 h or 4 h. For imaging flow cytometry (ImageStream), cells were harvested, washed with PBS fixed with 2% PFA for 10 min at RT and permeabilized with 90% (v/v) methanol for 20 min on ice. Primary antibody staining was a 1 : 50 dilution of NFAT-specific antibody (CellSignalling) and secondary anti-rabbit Alexa405 for 1 h at 4°C with washing steps in between. Nuclei were stained with a 1 : 2000 dilution of DRAQ5. Cells were then transferred in FACS tubes and measured with an Amnis ImageStream X Mark II imaging flow cytometer (Merck-Millipore). A software-calculated mask based on DRAQ5 fluorescence was created to identify the nucleus in imaged T cells. The NFAT (Alexa405) fluorescence intensity within the nucleus of all measurements was then quantified and plotted as histograms.

### 4.10. Software and statistical analysis

For confocal microscopy, image analysis was performed using Volocity v. 6.2 (PerkinElmer) and ImageJ. Figures were generated with CorelDrawX4 graphics suite, Microsoft PowerPoint and GraphPad Prism v. 5.0 software. Statistical calculations were also done with GraphPad Prism v. 5.0. The respective statistical test used is indicated in each figure legend.

## Supplementary Material

Supplemental Data Set 1

## Data accessibility

The datasets supporting this article have been uploaded as part of the electronic supplementary material.
