# Supplementary material for: Virion encapsidated HIV-1 Vpr induces NFAT to prime non-activated T cells for productive infection
Source: Open Biol. 2016 Jul 6;6(7):160046. doi: 10.1098/rsob.160046 (PMC4967821; doi:10.1098/rsob.160046)
Supplement: Supplemental Data Set 1 [file rsob160046supp1.pdf]

Supplemental Material:

Virion encapsidated HIV-1 Vpr induces NFAT to prime non-activated T cells for productive infection.

Kristin Höhne[1,2], Ramona Businger[4], Anouk van Nuffel[3], Sebastian Bolduan[1], Herwig Koppensteiner[1], Ann Baeyens[3], Jolien Vermeire[3], Eva Malatinkova[5], Bruno Verhasselt[3,#], Michael Schindler[1,2,4,#]

[1]Institute of Virology, Helmholtz Zentrum München, German Research Center for Environmental Health, Neuherberg, Germany

[2]Heinrich Pette Institute, Leibniz Institute for Experimental Virology, Hamburg, Germany

[3]Department of Clinical Chemistry, Microbiology and Immunology, Ghent University, Ghent, Belgium

[4]Institute of Medical Virology and Epidemiology of Viral Diseases, University Hospital Tübingen, Tübingen, Germany

[5]HIV Translational Research Unit, Department of Internal Medicine, Ghent University, Ghent, Belgium

# Corresponding authors:

Michael Schindler, michael.schindler@med.uni-tuebingen.de

Bruno Verhasselt, bruno.verhasselt@ugent.be

Supplemental Figures S1-S5

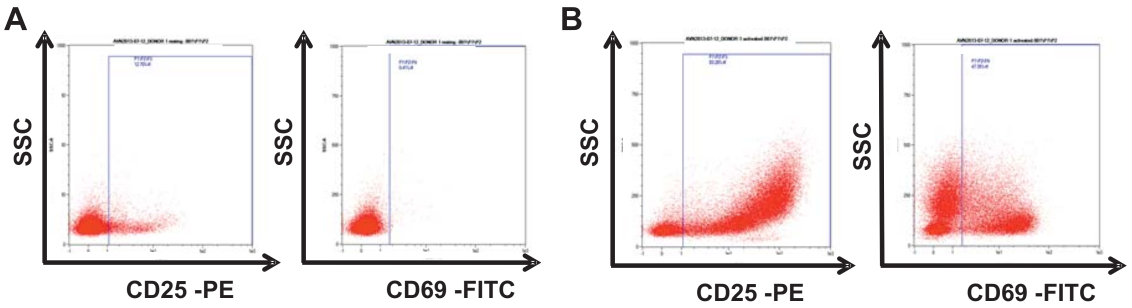

Supplemental Figure 1: Analyses of T cell activation markers in cultured CD4+ T cells. Presence of activation markers CD25 and CD69 were assessed by cell surface antibody FACS staining after 3 days of CD4+ T cell culture in IL-2 alone (A) or in IL-2 and PHA (B).

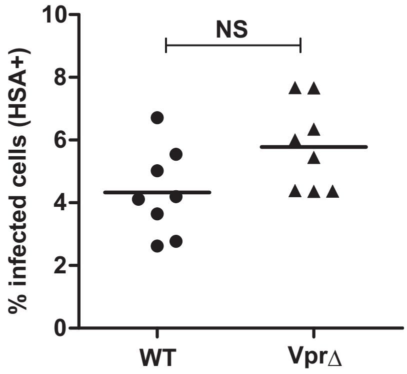

Supplemental Figure 2: Productive WT and VprΔ HIV-1 infection of primary prestimulated CD4+ T cells. Primary CD4+ T cells were stimulated with PHA/IL-2 for 3 days and subsequently infected with equal p24 amounts of HIV-1 WT or the VprΔ variant. Another 3 days later the amount of infected cells was determined by HSA staining and flow cytometry. Results show the mean of at least 8 independent experiments with CD4+ T cells from different donors and independent virus stocks.

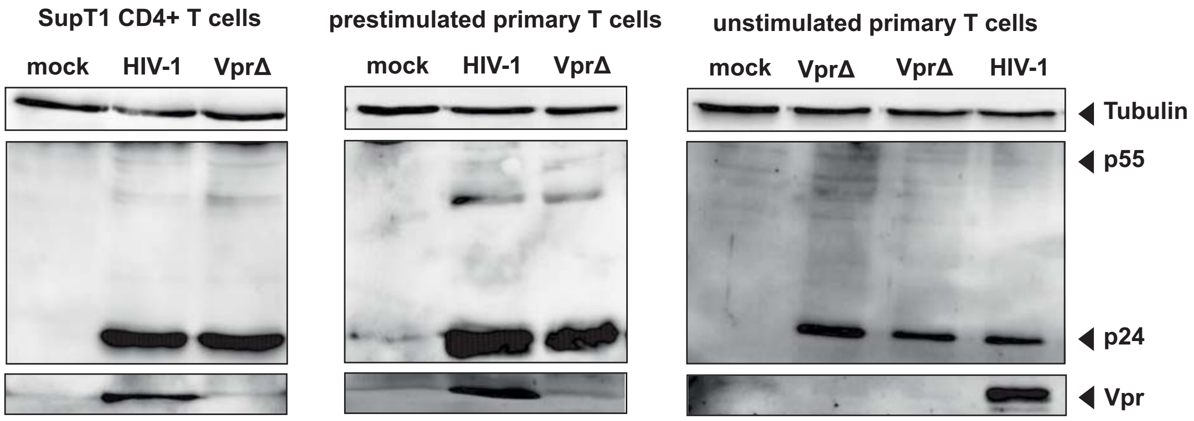

Supplemental Figure 3: Vpr does not affect the ability of HIV-1 to enter T cells as measured by capsid delivery. Western blots show staining for tubulin, p24 (Gag) and Vpr as indicated, on the cell line SupT1, peripheral blood CD4+ T cells (PHA/IL2 stimulated for 3 days; prestimulated primary T cells) or left untreated (unstimulated primary T cells), infected as described in methods. Per cell type, all were infected with equal amounts of virus, measured by p24 antigen concentration. Mock: uninfected, HIV: pBR NL4-3; ΔVpr: pBR NL4-3 ΔVpr. For unstimulated primary T cells left ΔVpr lane: pBR NL4-3 ΔVpr and right ΔVpr lane: NL4-3-IRES-HSA ΔVpr. 24 hours after infection, cells were washed and lysates were prepared for WB analysis. Representative experiments are shown.

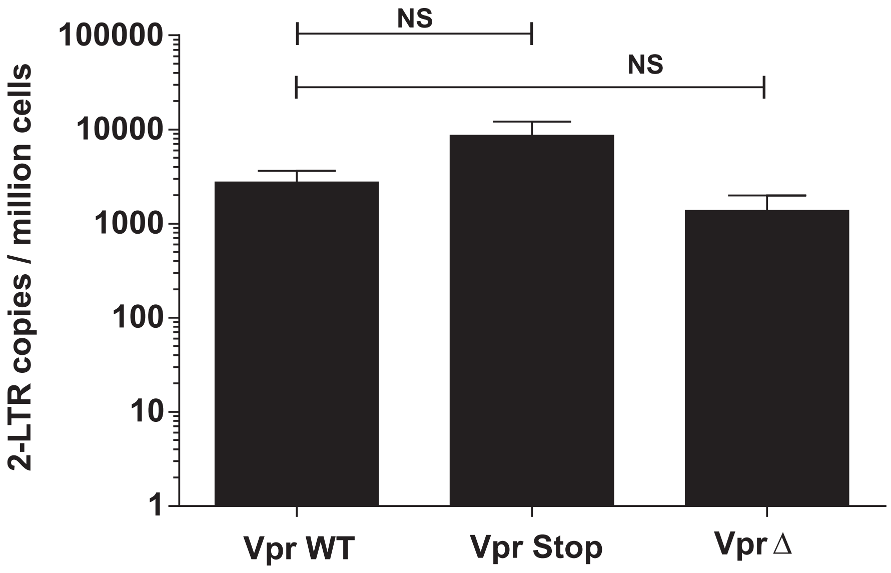

Supplemental Figure 4: Vpr's effect on productive infection in primary CD4+ T cells is not the result of defective nuclear import. Primary CD4+ T cells were infected as before (compare Fig. 1A). 3 days post-infection cells were collected and DNA was extracted. 2-LTR circles were quantified by qPCR. Results show the mean of 4 independent experiments carried out on 4 individual donors. No statistical difference was found (Kruskal-Wallis test with Dunn's correction).

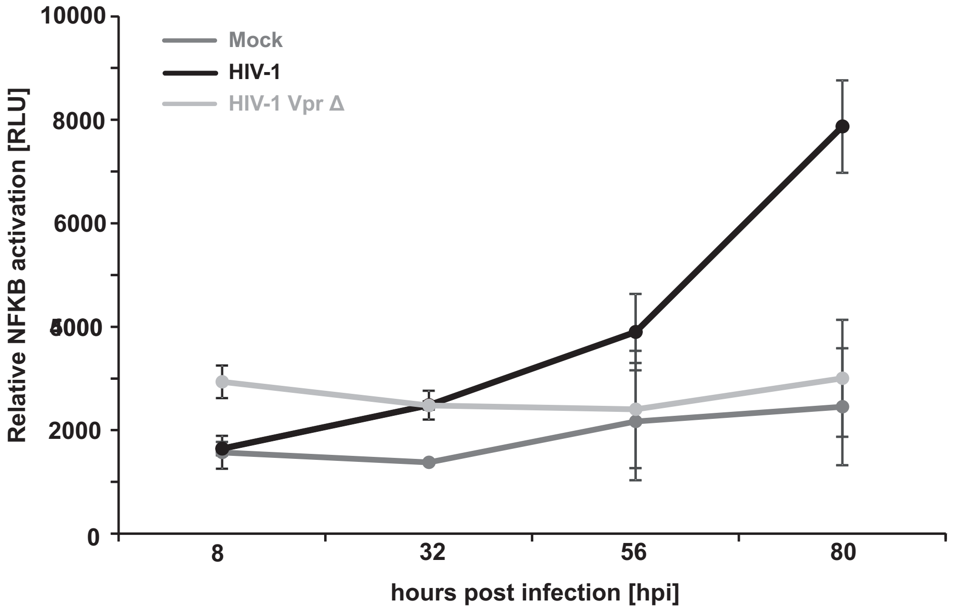

Supplemental Figure 5: Vpr stimulates NFKB at a late time point in the HIV-1 replication cycle. 293 cells expressing luciferase under control of the NFKB promoter were infected with HIV-1 similar to the experiment described in Fig. 2A and stimulated with 10 ng TNFα eight hours before each measurement. The experiment shows the mean and SD of three independent infections.
